# Exceptionally Preserved Cambrian Trilobite Digestive System Revealed in 3D by Synchrotron-Radiation X-Ray Tomographic Microscopy

**DOI:** 10.1371/journal.pone.0035625

**Published:** 2012-04-25

**Authors:** Mats E. Eriksson, Fredrik Terfelt

**Affiliations:** Department of Geology, Lund University, Lund, Sweden; Institut de Biologia Evolutiva - Universitat Pompeu Fabra, Spain

## Abstract

The Cambrian ‘Orsten’ fauna comprises exceptionally preserved and phosphatised microscopic arthropods. The external morphology of these fossils is well known, but their internal soft-tissue anatomy has remained virtually unknown. Here, we report the first non-biomineralised tissues from a juvenile polymerid trilobite, represented by digestive structures, glands, and connective strands harboured in a hypostome from the Swedish ‘Orsten’ fauna. Synchrotron-radiation X-ray tomographic microscopy enabled three-dimensional internal recordings at sub-micrometre resolution. The specimen provides the first unambiguous evidence for a J-shaped anterior gut and the presence of a crop with a constricted alimentary tract in the Trilobita. Moreover, the gut is Y-shaped in cross section, probably due to a collapsed lumen of that shape, another feature which has not previously been observed in trilobites. The combination of anatomical features suggests that the trilobite hypostome is functionally analogous to the labrum of euarthropods and that it was a sophisticated element closely integrated with the digestive system. This study also briefly addresses the preservational bias of the ‘Orsten’ fauna, particularly the near-absence of polymerid trilobites, and the taphonomy of the soft-tissue-harbouring hypostome.

## Introduction

The ‘Orsten’ fauna was serendipitously discovered in the mid-1970s by micro-palaeontologist Klaus Müller when he dissolved Cambrian limestone concretions from south-central Sweden in search of conodonts [1]. This fauna primarily includes exceptionally preserved microscopic arthropods and has provided significant insights into early Phanerozoic metazoan evolution [2]–[5]. The ‘Orsten’ type phosphatisation involves encrustation and impregnation of external layers of animals during early diagenesis, producing a pristine three-dimensional fossil preservation. Intriguingly, this kind of preservation is restricted to complete or partial animals with a size less than 2 mm [5].

**Figure 1:**
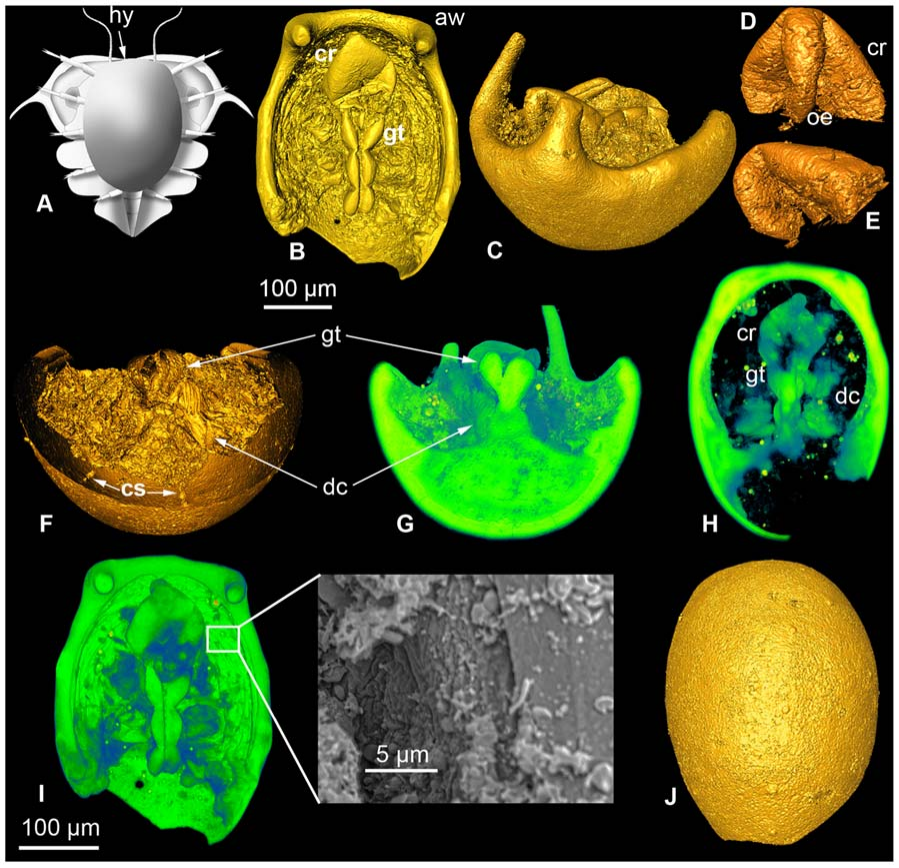
Trilobite reconstruction, SRXTM 3D renderings and SEM images. (A) Hypothetical reconstruction of the ventral side of an early meraspid stage of *Sphaeropthalmus*? sp. (B–J) 3D rendering of a SRXTM dataset of the hypostome with digestive system (LO 11348t; specimen stored at the Department of Geology, Lund University, Sweden); isosurface (B–F, J) and semi-transparent voltex images (G–I). (B–C) Interior and oblique lateral view. (D–E) Isolated oesophagus and crop in different views. (F) Anterior cross-section. (G) Posterior view, transect through mid-part. Note the Y-shaped gut in the central part of the image. (H) Interior view, transacted from below and above. (I) Interior view with SEM close-up of internal lining. (J) Exterior view, showing minute tubercles. Abbreviations: aw, anterior wing; cr, crop; cs, connective strand; dc, digestive cecum; oe, oesophagus; gt, gut tract; hy, hypostome.

In this study, we report the discovery of trilobite digestive structures, harboured in a hypostome (Figure 1), from the Swedish ‘Orsten’ fauna. This remarkable specimen was recovered from Cambrian (Furongian) strata of the Backeborg locality at Mt. Kinnekulle, south-central Sweden [5]. The conventional record of shelly fossils in the Furongian of Sweden is dominated by olenid trilobites [6], [7], commonly occurring in bewildering numbers in shales and limestones. In light of this, together with the fact that substantial amounts of limestone of this age has been dissolved in the search for exceptionally preserved fossils [8], it is puzzling that until now not a single polymerid trilobite has been reported with preserved non-biomineralised parts from the ‘Orsten’ *Konservat-Lagerstätte*.

Although trilobite guts preserved in three dimensions have been previously reported (e.g. [9]–[14]) our knowledge of the internal anatomy, including the digestive system, is limited due to the poor preservation potential of such structures. Our specimen was analysed using scanning electron microscopy (SEM) and synchrotron-radiation X-ray tomographic microscopy (SRXTM) [15], [16] which enabled internal recordings at sub-micrometre resolution. SRXTM allowed detailed three-dimensional anatomy of the trilobite digestive system to be revealed, facilitating comparison with extant arthropods and potentially adding phylogenetically important characters. Moreover, the specimen offers an opportunity to interpret important aspects of the hypostome function.

## Results and Discussion

### Hypostome and soft-tissue anatomy

The 300 µm long, deeply convex hypostome (LO 11348t) possesses two prominent anterior wings (Figures 1B, C; Video S1). Based on comparative morphology, size, and co-occurring fossils, the hypostome is tentatively assigned to an early ontogenetic stage of the olenid trilobite *Sphaerophthalmus* of the Furongian *Ctenopyge affinis* Zone [7]. The trilobite hypostome is a ventral sclerite that was attached to the dorsal exoskeleton in the anterior region of the glabella. In adults, it covered an area corresponding to the anterior half (or more) of the glabella, but it could cover a much larger portion of the body in larvae [17]–[20], acting as protective shield comparable to the labrum of some recent crustacean larvae [21] (Figure 1A).

The most conspicuous feature of the specimen is the centrally located gut, comprising an oesophagus terminating in a crop, which in turn continues into a sagitally folded and transversally constricted gut tract (Figure 1B). These constrictions are interpreted as circular muscles, exerting sucking pressure on the food through the digestive tract by means of peristaltic contractions. Similar circular muscles are present in the gut tract of extant arthropods [22], [23]. Alternatively, the constrictions observed in our specimen could represent metamerically ordered, extrinsic muscle attachment sites from the gut to the visceral side of the head (cf. [23]). The oesophagus is sharply curved beneath the crop and terminates posteroventrally, resulting in an over-all J-shaped structure (Figures 1D, E, Video S1). This unambiguously demonstrates that the anterior gut of trilobites was J-shaped (cf. [14]), an issue that has been hypothesised at least since Richter [24]. The specimen also provides the first unambiguous evidence for the presence of a crop and a constricted alimentary tract in the Trilobita (cf. [12], [25]). Moreover, the gut section posterior to the crop has a Y-shaped cross section (Figure 1G, Video S2), probably due to a collapsed Y-shaped gut lumen prior to phosphatisation. This feature has not previously been observed in the Trilobita.

Cisne [9]–[11] analysed several specimens of the trilobite *Triarthrus eatoni* in detail and, by using an X-ray apparatus, was able to detect several pyritised, anatomical features (exoskeleton, skeletomuscular and digestive systems). Although the radiographs of Cisne [10], [11] clearly show the appendages, the digestive system is considerably less conspicuous and rather difficult to interpret. Nonetheless, based on those radiographs he constructed schematic interpretative drawings that showed parts of the *T. eatoni* digestive system. He postulated a stomach connected to an intestine located in the anterior head region. Moreover, he interpreted the stomach as connected to an oesophagus, which in turn made a posteriorly directed loop and terminating in the mouth; hence resulting in an overall J-shaped gut. This is similar to what we observe in the juvenile ‘Orsten’ hypostome, however, we cannot evaluate the exact position of the mouth (except for the fact that it was posteriorly directed) relative to the whole trilobite body.

**Figure 2:**
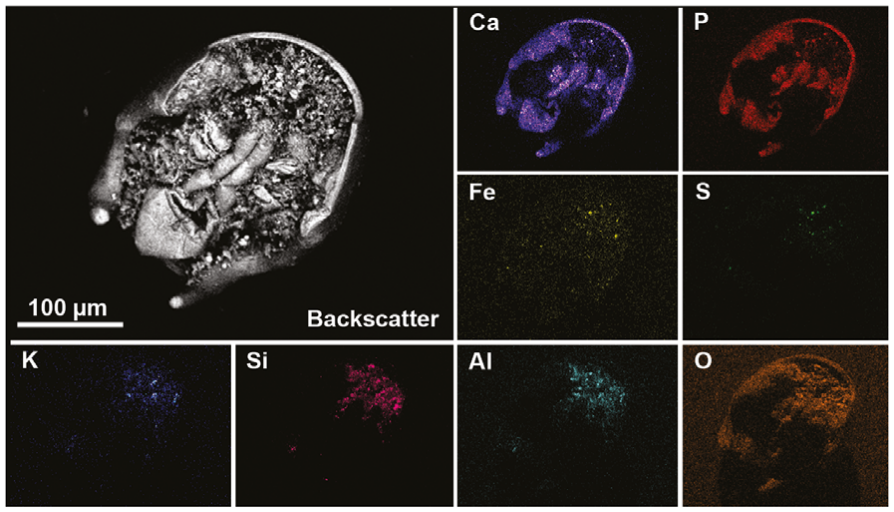
Backscatter (SEM) image and elemental maps of hypostome (LO 11348t). Relative amount of an element is indicated by the brightness; the brighter the colour, the higher the level of a certain element. The internal organs and hypostome sclerite are completely phosphatised whereas the adhering matrix consists of aluminium silicate minerals and some pyrite.

Biserially to the gut tract in our specimen are at least two (possibly three) pairs of folded digestive caeca (Figures 1F–H, 2, Video S2), comparable to the digestive glands seen in extant remipedian crustaceans [14]. The digestive caeca are attached to the lower part of the gut (Figure 1G). In extant arthropods, digestive caeca usually attach to the midgut, either dorsally, laterally and/or ventrally (e.g. [26], [27]). Although it has long been thought that the digestive caeca in trilobites were laterally attached to the tract, Lerosey-Aubril *et al.* [14] recently showed that they insert dorso-laterally. By contrast, in our specimen the digestive caeca attach ventrally.

The combination of a crop and digestive caeca is unique. Chatterton *et al.* [12] and Lerosey-Aubril *et al.* [25] hypothesised that two different types of digestive systems were present in trilobites (excluding agnostoids). One is characterised by a simple tract, which shows no clear evidence of a differentiated foregut into a crop and is flanked laterally by metamerically paired digestive caeca. The latter differs from the former in lacking digestive caecae but having a crop [25]. Thus, the *Sphaerophthalmus*? specimen provides compelling evidence that both these structures could occur in a trilobite. However, the specimen is from a very young individual, whereas previous observations of trilobite digestive systems were on adults. It is possible that the size of the crop, relative to the intestine behind, decreased during ontogeny, making it difficult to distinguish in adults.

The internal hypostome wall is covered by longitudinally folded and bilateral symmetrical structures with an anastomosing pattern at microscopic scale (Figures 1B, I). These structures may represent glands associated with the digestive system or they could be soft-tissues covering the entire visceral side of the hypostome.

The exterior hypostome surface has minute tubercles (Figure 1J). The corresponding interior view of these tubercles have attached string-like structures protruding towards the central inner part (Figure 1F), suggestive of connective tissues/muscles attaching the hypostome to the underside of the glabella and to the digestive tract. This supports the view that the hypostome was connected to the anterior gut by connective tissues/muscles (e.g. [24], [25], [28]). Collectively, these anatomical features suggest that the hypostome was not simply a protective sclerite and/or scooping device, but an elaborate component closely integrated with the digestive system.

There has been controversy regarding the definition of the labrum (e.g. [2], [29], [30]) and the view that the hypostome and the labrum are of different phylogenetic origin (see [2]) may well be true. However, the presence of connective tissues and putative glands in the specimen at hand suggests that the trilobite hypostome is functionally analogous to the euarthropod labrum *sensu* Maas *et al.* [2], which includes chemoreceptors, muscles and secretory glands [2], [31].

### Preservation

Elemental mapping of the specimen revealed ‘Orsten’-type phosphatisation, with some clay minerals in the adhering matrix (Figure 2). Phosphatisation of the ‘Orsten’ fauna is generally thought to have involved early diagenetic encrustation/impregnation of the external layers of animals (e.g. [2], [5], [32]) with the source of phosphorous being coprolites [32]. The specimen described here is a unique case of ‘Orsten’ preservation as it represents the only polymerid trilobite reported so far with internal organs. The preservation of the soft-tissues is intriguing. Butterfield [33] showed that even labile structures, such as midgut glands, can become phosphatised from internal sources and that an accentuated chemical reactivity enhanced their preservation potential by providing a focus for early diagenetic mineralisation. For our specimen it seems plausible that phosphate-rich fluids entered the body through the mouth permitting the digestive system to be preserved (although phosphatisation of other tissues such as the hypostome sclerite suggests also external sources). Alternatively, the trilobite was disarticulated immediately after death and the detached hypostome, including the digestive structures, was submerged in phosphate-rich fluids. Regardless of which, the phosphatisation process must have been rapid in order for these labile structures to become preserved. A commonly invoked parameter for phosphatisation is bacterial activity (e.g. [14] and references therein), however, our SEM analyses did not reveal any evidence of such processes.

The fact that the hypostome described herein belonged to an early juvenile is in accordance with the view that whatever the processes involved in the soft-tissue phosphatisation in the ‘Orsten’ fauna, these could only permit the preservation of small organisms/biological structures (e.g. [2], [5]). The reason for the extreme rarity of phosphatised tiny polymerid trilobites (or parts of them) in the ‘Orsten’ fauna is still elusive. Albeit speculative, different life strategies between polymerids and other common ‘Orsten’ taxa offer a possible explanation. For example, benthic organisms, and infaunal ones in particular, would presumably have a greater potential for rapid, post-mortem entombment and phosphatisation than planktonic and nektonic ones. Some polymerid trilobite taxa were probably planktonic during their earliest life phases (e.g. [34], [35]), which could explain the near-absence of phosphatised trilobite larvae in the ‘Orsten’ *Lagerstätte*.

## Materials and Methods

A small ‘Orsten’ slab (*c.* 50 g) was digested in buffered acetic acid [36]. The remaining residue was rinsed through a 63 micrometre sieve cloth and washed into a glass vial with de-ionized water. The residue was studied under a binocular light microscope and the specimen was hand-picked using a fine brush.

Synchrotron radiation X-ray tomographic microscopy (SRXTM) was performed at the TOMCAT beamline of the Swiss Light Source, Paul Scherrer Institute, Switzerland [15], [16]. Our technical set-up is similar to that of Alwmark *et al.* [16]. The specimen was placed in a thin-walled, low X-ray scattering, capillary glass tube with an outer diameter of 500 µm, a wall thickness of 10 µm, and a height of 80 mm. The base of the capillary tube was first filled with 10–15 glass beads with a diameter of 25–30 µm. The capillary was subsequently mounted on sample holders using melted bees' wax and the uppermost part of the empty capillary was broken off. In order to optimize the contrast, the beam energy was set to 12 keV. The X-ray radiation transmitted by the sample was converted into visible light by a 20 µm thick Ce-doped LuAG scintillator screen (Crytur, Turnov, Czech Republic). Projection images were magnified by microscope optics and digitized by a high-resolution CCD camera with a 2048×2048 pixel chip and a pitch of 7.4 µm (PCO2000; PCO GmbH, Kelheim, Germany). The optical magnification was set to 20×, resulting in cubic voxels of 0.37 µm in the reconstructed data sets. For each scan, 1501 projections were acquired along with starting dark and flat field images. The exposure time was 200 ms for each projection, thus the complete data set was acquired in approximately 15 min. The tomographic reconstructions were performed on a 60-node Linux PC cluster using a highly optimised routine based on the Fourier transform method and a gridding procedure [37]. The resulting tif micro-tomograms, or slices, were imported and rendered, using the commercial software Voxler2, into 3D-images that could be studied from every angle and virtually cut into different planes.

For elemental mapping, the uncoated specimen was mounted on a sample stub and analysed in low vacuum in a Hitachi S-3400N scanning electron microscope (SEM). It was performed with an energy dispersive spectrometer (Inca X-sight, Oxford instruments) with a Si-detector. The acceleration voltage was 15 keV and the beam current ca. 2 mA.

## Supporting Information

Video S1 **Video clip showing the hypostome with digestive system (LO 11348t).** External morphology (isosurface).
(MP4)

Video S2 **Video clip showing the hypostome with digestive system (LO 11348t).** Semi-transparent (voltex) with clipping plane transecting the specimen from posterior to anterior.
(MP4)
